# Daily Consumption of α-Linolenic Acid Increases Conversion Efficiency to Eicosapentaenoic Acid in Mice

**DOI:** 10.3390/nu16101407

**Published:** 2024-05-07

**Authors:** Saori Watabe, Wataru Tanaka, Hiroyuki Sakakibara, Daigo Yokoyama

**Affiliations:** 1 Graduate School of Agriculture, University of Miyazaki, 1-1 Gakuen-Kibanadai Nishi, Miyazaki 889-2192, Japan; sakuranbo80101016@gmail.com (S.W.); w.tanaka004159@gmail.com (W.T.); 2 Graduate School of Agricultural Science, Kobe University, Kobe 657-8501, Japan

**Keywords:** ω-3 fatty acid, mouse, daily consumption, eicosapentaenoic acid, α-linolenic acid

## Abstract

To maintain a beneficial concentration of eicosapentaenoic acid (EPA), the efficient conversion of its precursor, α-linolenic acid (α-LA), is important. Here, we studied the conversion of α-LA to EPA using ICR and C57BL/6 mice. A single dose of perilla oil rich-in α-LA or free α-LA had not been converted to EPA 18 h following administration. The α-LA was absorbed into the circulation, and its concentration peaked 6 h after administration, after which it rapidly decreased. In contrast, EPA administration was followed by an increase in circulating EPA concentration, but this did not decrease between 6 and 18 h, indicating that the clearance of EPA is slower than that of α-LA. After ≥1 week perilla oil intake, the circulating EPA concentration was >20 times higher than that of the control group which consumed olive oil, indicating that daily consumption, but not a single dose, of α-LA-rich oil might help preserve the physiologic EPA concentration. The consumption of high concentrations of perilla oil for 4 weeks also increased the hepatic expression of *Elovl5*, which is involved in fatty acid elongation; however, further studies are needed to characterize the relationship between the expression of this gene and the conversion of α-LA to EPA.

## 1. Introduction

ω-3 polyunsaturated fatty acids (PUFAs) have a characteristic structure that involves a double bond three atoms away from the terminal methyl group, and they are widely distributed in nature. The principal physiologic ω-3 fatty acids are α-linolenic acid (α-LA, 18:3 *n*-3), eicosapentaenoic acid (EPA, 20:5 *n*-3), and docosahexaenoic acid (DHA, 22:6 *n*-3). α-LA is principally obtained from plant oil mixtures, such as perilla and linseed oil, whereas EPA and DHA are obtained from fish and other marine organisms [1,2]. Interestingly, EPA and DHA have been reported to have beneficial and protective effects against several diseases, including obesity, diabetes, chronic obstructive pulmonary disease, colorectal cancer, heart failure, and other cardiovascular diseases [3,4,5,6,7], whereas α-LA does not have the same health benefits [8]. Because humans cannot efficiently synthesize EPA or DHA, it is important that adequate amounts of fish and fish-oil products are consumed [8]. However, the intake of EPA and DHA in fish and fish oil is often insufficient, at least in part because the amounts of EPA and DHA in fish are highly variable [9]. This is because EPA and DHA are not synthesized by fish but instead accumulate in their bodies as a result of the consumption of EPA- and DHA-rich cold-water algae; therefore, the diet of the fish has a large effect on their levels of EPA and DHA [10]. In addition, the taste and smell of fish are not universally appealing, and knowledge of the deleterious effects on health of the heavy metals in fish may also result in inadequate EPA and DHA intake in fish oil [9]. Therefore, to obtain the benefits of ω-3 PUFAs, such as EPA and DHA, it would be useful to supplement the intake of these fatty acids from other food materials in addition to fish and fish oil.

Mammals such as humans and mice do not have the enzymes necessary to synthesize α-LA from palmitic acid; therefore, the daily ingestion of ω-3 PUFAs is necessary [11,12,13]. However, elongases and desaturases, which are the enzymes responsible for the conversion of α-LA to EPA and DHA, are present in mammals [14]. Therefore, although humans cannot synthesize EPA and DHA de novo, they can be generated from α-LA, which is a shorter-chain ω-3 PUFA. Hence, the daily consumption of α-LA rich food components, such as perilla and linseed oils, represents an alternative means of maintaining physiologic concentrations of EPA and DHA. However, not all the α-LA absorbed into the body is converted to EPA and DHA with conversion rates estimated to be 0.3–10% [8,15,16,17]. Although this conversion efficiency is not high, we hypothesized that the large variation in the conversion rates calculated during previous studies may indicate that ingested α-LA may be more efficiently converted to EPA and DHA under particular conditions. Therefore, in the present study, we conducted an oral lipid tolerance test in a mouse model using α-LA or α-LA rich perilla oil [18] to evaluate the conversion efficiency of α-LA to EPA. Olive oil with low α-LA amount was used as a comparative oil [19]. Following, the conversion efficiency was also evaluated when the mice were fed ad libitum a diet containing either perilla oil or olive oil for 1 or 4 weeks.

## 2. Materials and Methods

### 2.1. Chemicals

EPA (95.0% purity) was purchased from Combi-blocks, Inc. (San Diego, CA, USA). α-LA (>98.0% purity), casein, soybean oil, l-cystine, and t-butylhydroquinone were purchased from Wako Pure Chemical Industries (Osaka, Japan). Cellulose, α-cornstarch, β-cornstarch, and sucrose were obtained from Oriental Yeast Co., Ltd. (Tokyo, Japan). Vitamin mix (AIN-93-VX) and mineral mix (AIN-93G-Mix) were from MP Biomedicals, LLC (Irvine, CA, USA). All other reagents were of the highest grade available.

### 2.2. Animal Experiments

#### 2.2.1. Institutional Approval of the Study Protocols

All the animal experiments were conducted with the approval of the Institutional Animal Experimentation Committee of the University of Miyazaki (approval no. 2020-011-3 and date of approval: 25 March 2022). The study was conducted in accordance with the Law Concerning the Humane Treatment and Management of Animals (law no. 105 of 1973), which defines animal experimentation as the use of animals for scientific purposes with consideration of the 3Rs.

#### 2.2.2. Animals

**Figure 1:**
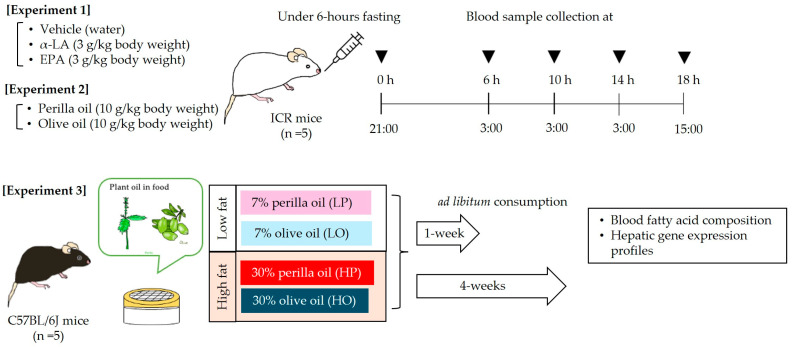
Schematic of the experimental design. See Table 1 for the compositions of the individual diets. α-LA, α-linolenic acid; EPA, eicosapentaenoic acid.

Twenty-five male ICR and forty C57BL/6 mice were purchased from Japan SLC (Shizuoka, Japan). They were provided ad libitum with deionized water and powdered AIN-93G (LS) diet, which included 7% soybean oil (Table 1); and they were housed at a temperature of 23 ± 2 °C, at a humidity of 55 ± 10%, and under a 12 h light/dark cycle (lights on at 09:00). After an acclimation period, the following three experiments were performed (Figure 1). The number of mice in each experimental group was set to 5 to reduce the number of experimental animals used and to allow for statistical analysis even if some mice unavoidably dropped out due to lacerations or other reasons. In order to reduce the number of animals used, only the vehicle solvent group in Experiment 1 was limited to three animals per group. In the three experiments conducted in this study, no mice dropped out during the experimental period. To minimize potential confounders, in Experiments 1 and 2, mice were assigned a dose number, the order of administration to the mice was randomized, and administration and blood sampling were performed without the practitioner knowing the type of drug administered. Similarly, in Experiment 3, blood samples and other procedures were performed without the researcher knowing which diet was consumed.

#### 2.2.3. Experimental Design

Experiment 1: α-LA or EPA was administered to mice using the oral lipid tolerance test protocol reported by Ochiai et al. with some modifications [20]. Briefly, 15 male ICR mice aged 8 weeks that were consuming the control LS diet were randomly allocated to three groups: an α-LA group (3 g/kg body), an EPA group (3 g/kg), and a water group (3 mL/kg). After 6 h of fasting, each of the fatty acids was orally administered at 21:00. Blood samples were then collected from a tail vein under isoflurane anesthesia (2.0%) 0 (before administration), 6, 10, 14, and 18 h following the administration. The samples were collected into heparinized hematocrit capillary tubes (EM MYSTAR Hematocrit Capillary Heparin Treatment, AZ ONE, Osaka, Japan), and then an aliquot (25 μL) was applied to butylated hydroxytoluene (BHT; an antioxidant)-treated filter paper, according to the manufacturer’s protocol for the Fatty Acid Methylation Kit (Nacalai Tesque, Inc., Kyoto, Japan). After drying in a desiccator, the fatty acids were isolated, methylated according to the manufacturer’s protocol, and then subjected to gas chromatographic analysis, as described below.

**Figure 2:**
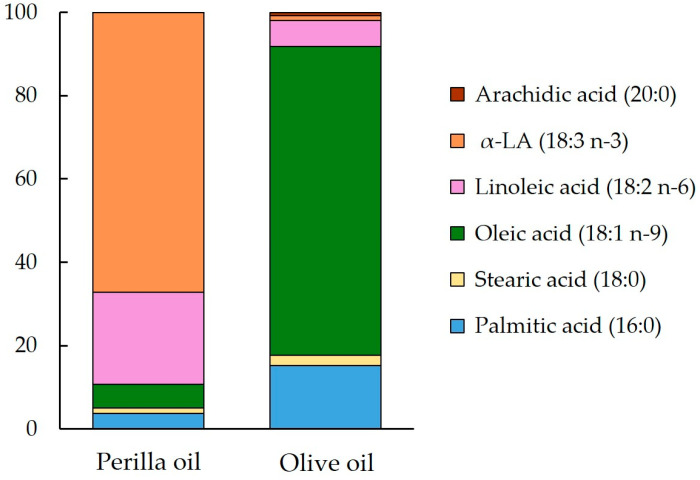
Fatty acid composition of the perilla and olive oils used in the study. α-LA, α-linolenic acid. See Section 2.3.1 for the method of measuring fatty acid composition.

Experiment 2: Ten male ICR mice aged 8 weeks that were consuming the control LS diet during the acclimation period were randomly allocated to two groups. The first group was administered perilla oil (a ω-3 PUFA-rich oil; 10 g/kg), and the second was administered olive oil (an ω-9 PUFA-rich oil; 10 g/kg). The fatty acid compositions of these oils were measured according to the method described Section 2.3.1. The results are shown in Figure 2. Both oils were administered at 21:00 after 6 h fasting; then, blood samples were collected from a tail vein under isoflurane anesthesia (2.0%) before and 6, 10, 14, and 18 h after the administration. Blood samples were collected into heparinized hematocrit capillary tubes, and then fatty acid samples were prepared as described above.

Experiment 3: Forty male C57BL/6J mice aged 8 weeks that were consuming control LS diet during the acclimation period were randomly allocated to four groups: a group fed a low-fat diet that contained 7% perilla oil (LP diet); a group fed a low-fat diet that contained 7% olive oil (LO diet); a group fed a high-fat diet containing 30% perilla oil (HP diet); and a group fed a high-fat diet containing 30% olive oil (HO diet) (Table 1). These groups consumed their diets ad libitum for 1 or 4 weeks. At the end of these periods, non-fasted blood samples were collected from an abdominal vein cava under isoflurane anesthesia (2.0%) at 15:00 into Capiject^®^II tubes (Terumo Medical Corporation, Tokyo, Japan). After being left to stand at room temperature for 30 min, the serum fraction was obtained by centrifugation at 3500× *g* for 90 s, and the samples were stored at −80 °C until further analysis. In addition, after being bled to death, liver samples were cut into approximately 3 mm cubes, immersed in RNALater^®^ (Sigma-Aldrich Co., LLC, St. Louis, MO, USA) and stored at 4 °C overnight, and then transferred to −20 °C until RNA was extracted.

### 2.3. Biochemical Parameters

#### 2.3.1. Circulating Fatty Acids

Fatty acid compositions of the BHT-treated blood, serum and oil samples were analyzed using a Fatty Acid Methylation Kit (Nacalai Tesque, Inc., Kyoto, Japan), according to the manufacturer’s protocol and the method used previously [21]. Specifically, fatty acids were extracted from 10 μL blood, 25 µL serum and 10 mg oil samples and methylesterified; then, the products were dissolved in 200 μL of hexane. These samples were analyzed using a gas chromatograph (GC-2014, Shimadzu Corporation, Kyoto, Japan) and a Supelco Wax^TM^ 10 capillary column (30 m long × 0.32 mm internal diameter × 0.25 μm film thickness; Sigma-Aldrich Co., LLC). The column oven temperature was increased from 170 to 225 °C, and a hydrogen flame ionization detector was used to detect the separated fatty acid methyl esters. The standard for methylesterified fatty acids (Supelco 37 Component FAME Mix) was purchased from Sigma-Aldrich Co., LLC.

#### 2.3.2. Quantitative Reverse Transcription (RT)-PCR

The hepatic gene expression of the mice was evaluated using a method modified from that used in our previous study [22]. Briefly, RNA was extracted using a QuickGene RNA tissue kit SII (RT-S2) and a QuickGene-Mini80 (Kurashiki Boseki, Osaka, Japan), according to the manufacturer’s instructions. The concentrations of the RNA samples obtained were determined using a Qubit^®^ RNA Assay kit and a Qubit^®^ 2.0 Fluorometer (Invitrogen, Carlsbad, MA, USA). The extracted RNA was then reverse-transcribed to cDNA using a PrimeScript^®^ RT reagent kit (RR037; Takara Bio Inc., Shiga, Japan) to a final concentration of cDNA of 400 ng/mL using the following conditions: 37 °C for 15 min, followed by heat inactivation at 85 °C for 5 s, and held at 4 °C. The products were stored at 4 °C.

Real-time quantitative PCR was performed using an AriaMx real-time PCR system (Version 3.1.1812.0301, Agilent Technologies, Inc., Santa Clara, CA, USA). The primers were obtained from Eurofins Genomics Ltd. (Tokyo, Japan), and the sequences are shown in Table 2. Each cDNA aliquot (0.5 µL) was added to 9.5 µL of PCR mixture consisting of 5 µL of Brilliant III Ultra-Fast Sybr^®^ Green Mater Mix (Agilent Technologies Inc.), 3.5 µL of DNase/RNase-free water, and 0.5 µL of each primer solution. The thermal cycling conditions were as follows: 50 °C for 2 min, initial activation at 95 °C for 10 min, then 45 cycles of denaturation at 95 °C for 30 s, annealing at 65 °C for 30 s, and extension at 72 °C for 30 s. The relative expression levels of the target genes were calculated using β-actin as the reference gene and the 2^−ΔΔCt^ method.

### 2.4. Statistical Analysis

Data are presented as mean ± standard deviation (SD) and were analyzed using two-way analysis of variance (ANOVA) in StatView for Windows (version 5.0, SAS Institute, Cary, NC, USA), followed by the Tukey–Kramer post hoc test. For within-group comparisons, the alpha value was set at 0.05.

## 3. Results

### 3.1. Pharmacodynamics of α-LA and EPA after a Single Administration

**Figure 3:**
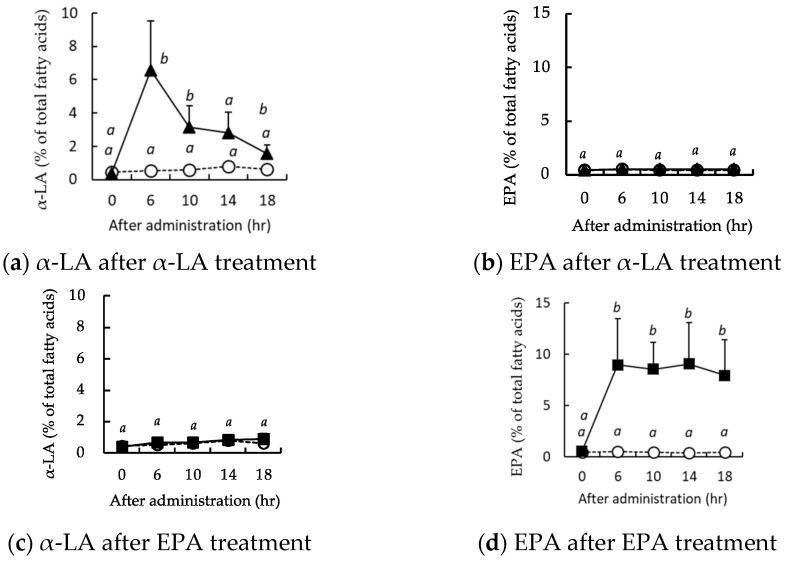
Changes in the circulating α-linolenic acid (α-LA) and eicosapentaenoic (EPA) concentrations of the mice after a single administration. The circulating concentrations of α-LA (**a**) and EPA (**b**) following α-LA administration, and the circulating concentrations of α-LA (**c**) and EPA (**d**) after EPA administration are shown. Vehicle treatment group (◯, *n* = 3); α-LA group (▲, *n* = 5); and EPA group (◼, *n* = 5). Data are mean ± SD. Differing alphabetical superscripts indicate significant differences (*p* < 0.05).

When α-LA was orally administered to mice after a 6 h fast, the circulating concentration of α-LA peaked after 6 h and then declined, returning to its initial concentration after 18 h (Figure 3a). In contrast, the EPA concentration did not change during the 18 h period following administration vs. the control group (Figure 3b). EPA administration did not cause a change in the circulating α-LA concentration (Figure 3c), but the EPA concentration was significantly higher after 6 h and remained at this level until 18 h following administration (Figure 3d).

### 3.2. Effects of a Single Administration of Perilla Oil or Olive Oil on EPA Biosynthesis

**Figure 4:**
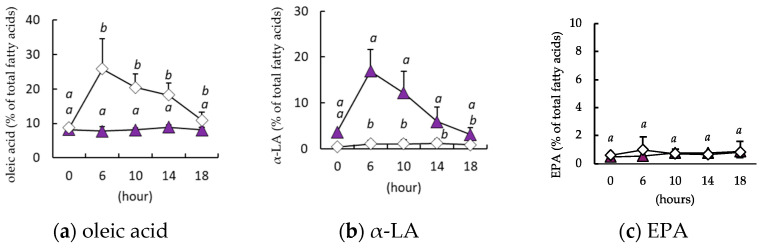
Changes in the circulating concentrations of α-linolenic acid (α-LA) and eicosapentaenoic (EPA) following perilla oil or olive oil administration. The circulating concentrations of oleic acid (**a**), α-LA (**b**), and EPA (**c**) are shown following the administration of perilla oil (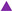) or olive oil (♢). Data are mean ± SD (*n* = 5). Differing alphabetical superscripts indicate significant differences (*p* < 0.05).

When olive oil or perilla oil was orally administered to mice after 6 h of fasting, the circulating concentrations of oleic acid (Figure 4a) and α-LA (Figure 4b), the major fatty acid components of each oil, peaked 6 h later and then declined, returning to their initial concentrations after 18 h. However, the circulating concentration of EPA did not change during the 18 h following the administration of either olive oil or perilla oil (Figure 4c).

### 3.3. Effect of the Daily Consumption of Perilla Oil or Olive Oil on EPA Biosynthesis

**Figure 5:**
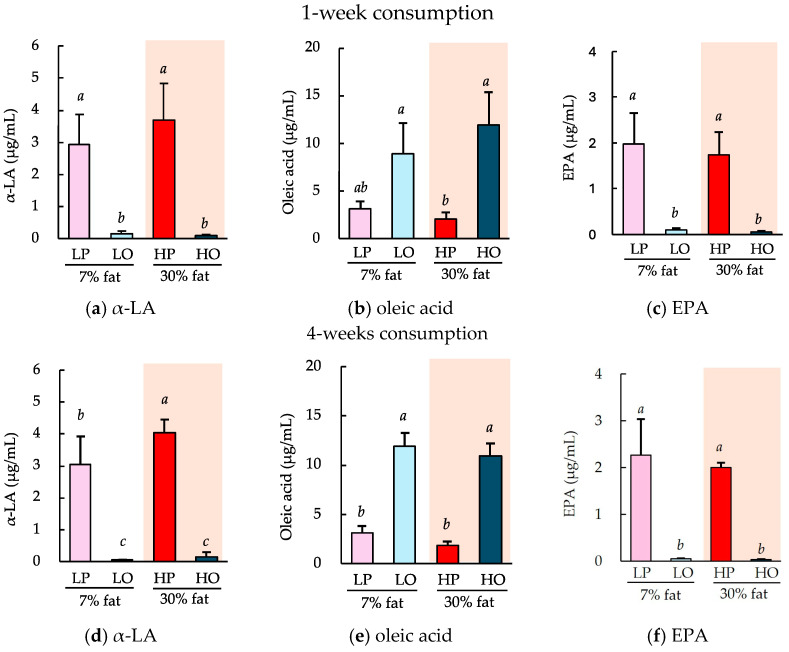
Effects of the ad libitum consumption of perilla oil or olive oil on the plasma α-LA, oleic acid, and EPA concentrations of the mice. After the ad libitum consumption of perilla oil or olive oil for 1 week (**a**–**c**) or 4 weeks (**d**–**f**), the plasma concentrations of α-LA (**a**,**d**), oleic acid (**b**,**e**), and EPA (**c**,**f**) were evaluated. The diets were either low-fat (7%) or high-fat (30%). LP, low-fat diet with perilla oil; LO, low-fat diet with olive oil; HP, high-fat diet with perilla oil; and HO, high-fat diet with olive oil. Data are mean ± SD (*n* = 5). Differing alphabetical superscripts indicate significant differences (*p* < 0.05).

**Figure A1:**
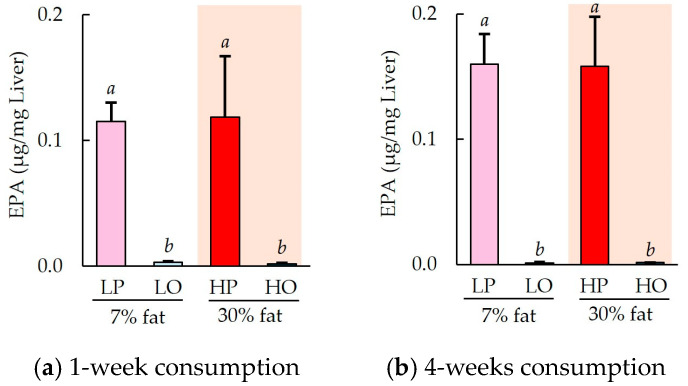
Effects of the ad libitum consumption of perilla oil or olive oil on the hepatic EPA concentrations of the mice. After the ad libitum consumption of perilla oil or olive oil for 1 week (**a**) or 4 weeks (**b**), the hepatic concentrations of EPA was evaluated. The diets were either low fat (7%) or high fat (30%). Data are mean ± SD (*n* = 5). Differing alphabetical superscripts indicate significant differences (*p* < 0.05).

There were no differences in the gains in body mass achieved among the groups consuming a 7% low-fat diet or a 30% high-fat diet containing perilla oil or olive oil as the principal lipid component for 1 or 4 weeks. Figure 5 shows the circulating concentrations of α-LA, oleic acid, and EPA. Regardless of whether the diets were consumed for 1 or 4 weeks, the major fatty acids in each oil, α-LA and oleic acid, were found to be present at high concentrations in the circulation. However, there was a significantly higher concentration of EPA in the perilla oil group (Figure 5c,f). These concentrations did not substantially differ according to the duration of consumption (1 or 4 weeks) or the amount of lipid in the diet (7% or 30%). In addition, hepatic EPA concentrations were also significantly higher in the perilla oil group regardless of the 1 or 4 weeks feeding period (Figure A1).

### 3.4. Effect of Daily Consumption of Each Oil on the Expression of Genes Encoding Fatty Acid Synthase

**Figure 6:**
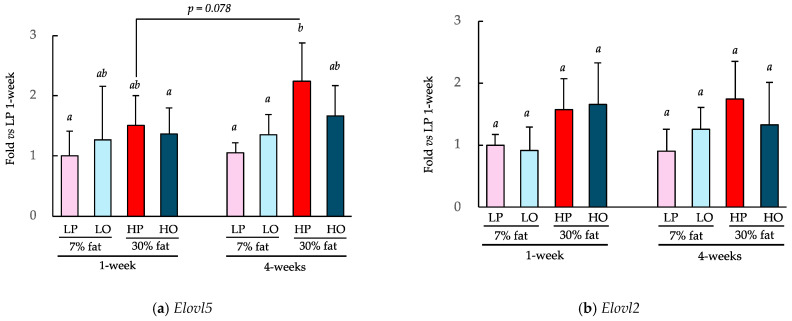
Effects of the ad libitum consumption of perilla oil or olive oil for 1 or 4 weeks on the hepatic gene expression of *Elovl2* and *5*. The diets were low-fat (7%) or high-fat (30%). LP, low-fat diet with perilla oil; LO, low-fat diet with olive oil; HP, high-fat diet with perilla oil; and HO, high-fat diet with olive oil. Data are mean ± SD (*n* = 5). Differing alphabetical superscripts indicate significant differences (*p* < 0.05).

The effects of consuming a 7% low-fat diet or a 30% high-fat diet containing perilla oil or olive oil as the principal lipid component for 1 or 4 weeks on the expression of key genes involved in fatty acid synthesis, *Elovl2* and *Elovl5*, are shown in Figure 6. The consumption of the HP diet for 4 weeks significantly increased *Elovl5* expression in the liver vs. the consumption of the LP diet over both 1 and 4 weeks (Figure 6a). The HO group showed a similar trend with *Elovl5* expression being 48% higher after 4 weeks of consumption (*p* = 0.078 with Student’s *t*-test). Four weeks of HP diet consumption resulted in a similar trend with respect to *Elovl2* expression (Figure 6b), but there were no differences between 1 and 4 weeks of consumption.

## 4. Discussion

The principal ω-3 PUFAs, EPA and DHA, which are abundant in fish oils and other seafoods, have been reported to have protective effects against several diseases, including obesity, diabetes, and cardiovascular disease [3,4,5,6,7]. However, it is difficult to obtain sufficient amounts of EPA and DHA to have these effects through the consumption of seafood alone [8]. Mammals cannot produce *n*-3 PUFAs because of the absence of Δ12-unsaturase and Δ15-unsaturase. Therefore, α-LA is an essential fatty acid that must be supplied in the diet [23]. In contrast, mammals can produce long-chain PUFAs, such as EPA and DHA, from α-LA [24]. Therefore, a greater consumption of plant-derived oils rich-in α-LA, such as perilla oil and linseed oil, may be a useful means of increasing the EPA and DHA concentrations. For example, Hussein et al. reported that people who consume α-LA-rich linseed oil for 12 weeks show a doubling of the circulating EPA concentration vs. the consumption of sunflower oil [16]. However, the physiologic conversion rates of α-LA to EPA and DHA are not high. According to the results of a clinical study conducted by Goyens et al., the conversion rate of ingested α-LA to EPA is 7%, and that for DHA is approximately 1% [25]. However, conversion rates of dietary α-LA to EPA and DHA of 0.3% and <0.01%, respectively, have also been reported [16]. Thus, dietary α-LA can be converted to EPA and DHA, but the amount converted varies. To identify an exploration for this, we have conducted a study of the effects of 1 and 4 weeks of consumption of a diet containing such oils using a mouse model.

When EPA, which is upstream in the metabolic pathway of α-LA and has a higher conversion rate than DHA [26], was administered orally as a single dose to mice, the circulating EPA concentration had increased 6 h later and remained at a similar concentration until at least 18 h. In contrast, a single oral administration of α-LA was associated with a peak after 6 h, after which the concentration decreased and had returned to the original value after 18 h. In general, in humans, after the ingestion of lipid-rich foods, the circulating concentrations peak after 3–6 h and return to their baseline concentrations 9–12 h later [27]. Similarly, in rodents, the baseline concentrations return after 8–12 h. Thus, the changes in circulating α-LA concentration following α-LA administration that occurred in the present study were similar to those previously reported [28]. This suggests that EPA may remain in the body for a longer period of time than other lipids, and similar results have been obtained when fish oil rich in EPA is administered orally [29].

We further evaluated the transformation of α-LA to EPA when administered in triglyceride rather than as a fatty acid, because fatty acids have been reported to have different bioavailability depending on the form of administration [30,31]. After a single administration of perilla oil to mice, in which 67.2% of the total lipid mass was α-LA, α-LA appeared in the circulation, as it did when free α-LA was administered, but the concentration of EPA was not affected. Following this, we fed mice a diet containing one of two lipid sources, α-LA-rich perilla oil or oleic acid-rich olive oil (containing very little α-LA). As a result, α-LA and oleic acid, the major fatty acids in each oil mixture, were detected in the blood, but there was no difference in the resulting concentrations between the 7% low-fat and 30% high-fat diets or between the consumption of the diets for 1 or 4 weeks. Interestingly, the circulating EPA concentration, which was not increased by a single dose, was >20-fold higher in mice consuming the perilla oil diet than in those consuming the olive oil diet, which lacked α-LA. This finding is similar to that of a previous study, which showed a 10-fold higher circulating EPA concentration in mice fed a diet containing perilla oil for 8 weeks compared to that associated with an olive oil-based diet [32]. DHA was below the lower limit of detection in both experiments in the present study. α-LA to DHA conversion was negligible, which is consistent with previous studies in which plasma DHA was below the lower limit of detection when fed α-LA [33,34]. No effects of the duration of intake or fat content of the diet were identified in this study, but the present results clearly indicate that continued daily intake, especially for more than 1 week, results in a significant increase in the physiologic conversion of α-LA to EPA. The reason why conversion to EPA was not identified following a single administration of α-LA but rather detected after 1 week of ad libitum consumption is currently unknown, but it is likely that the rate of conversion of the consumed α-LA to EPA is probably low after the single administration. However, the generated EPA in the body persists longer than other fatty acids, including α-LA, and therefore it may gradually accumulate if the supply of substrate continues. However, once the concentration exceeds a certain level, further accumulation of EPA may not occur with continued consumption. EPA may be difficult to digest and absorb, because it is relatively poor substrate for pancreatic lipase [35], but the mechanism of this is unknown. Thus, in the future, it will be necessary to elucidate why EPA administered as a free fatty acid remains in the circulation for such a long period of time.

α-LA is physiologically converted to EPA and DHA by desaturases and elongases [14,23,24]. Specifically, α-LA is converted to EPA by the action of δ-6 desaturase *Fads2*, the elongase *Elovl5*, and then δ-5 desaturase *Fads1*. EPA is further converted to docosapentaenoic acid (DPA) by the elongase *Elovl2* and then to DHA by *Elovl2* and *Fads2*. Therefore, we measured the expression of *Elovl5* and *Elovl2*, which are two key enzymes in these metabolic pathways. HP diet consumption for 4 weeks significantly increased the expression of hepatic *Elovl5* compared to that associated with the LP diet or LO diet. *Elovl5* expression is known to be induced by feeding, and by fat consumption in particular [36,37], and the effects of diet on *Elovl5* in the present study are consistent with these findings. However, because there was no difference in the expression of *Elovl5* between the LP and LO groups, it appears that daily consumption of an α-LA-rich diet does not affect the expression of *Elovl5*.

By conducting the present study, we found the following novel points: a single administration of α-LA is not physiologically converted to EPA; the physiological clearance of EPA is longer than that of α-LA; the conversion of α-LA to EPA is observed by the daily consumption of α-LA-rich oil such as perilla oil; its period is at least one week; and the daily consumption of α-LA increases *Elovl5* expression in the liver. On the other hand, we recognized that our study has several weaknesses. Specifically, the number of animals in each group was small (5 mice). Although this was enough for statistical analysis, the data, including gene expression analysis, showed large variabilities, and therefore a larger number of animals, for example 8–10 mice per each group, could have provided a clearer effect. Additionally, ad libitum consumptions were set at 1 and 4 weeks. Based on the results, we concluded that at least 1 week was required for conversion of α-LA to EPA. However, we considered that a more definite period of consumption could have been mentioned by conducting the analysis over 2–6 individual days. Furthermore, because this study focused on the conversion to EPA, it did not analyze other ω-3 PUFAs such as DHA. We will conduct further research to cover these weak points.

## 5. Conclusions

In the present study, a single administration of α-LA did not cause an increase in EPA concentration by 18 h following administration regardless of the administration form. However, after ≥1 week of perilla oil rich in α-LA intake, the circulating EPA concentration was >20 times higher than that in the olive oil-consuming group containing little α-LA. In this study, only perilla oil was used as a source of α-LA-rich oil, but humans do not usually use only one oil as their sole source of lipids. However, the consumption of oils containing α-LA in humans, such as flaxseed oil, margarine, and canola oil, increases plasma concentrations of EPA [34], and therefore our results imply that the daily consumption of an α-LA-rich oils might help maintain a beneficial EPA concentration. Furthermore, the consumption of high concentrations of perilla oil for 4 weeks increases the expression of *Elovl5* in the liver; however, further studies are needed to characterize the relationship between this effect and the physiologic conversion of α-LA to EPA.

## Figures and Tables

**Table 1:** Compositions of the AIN-93G-based experimental diets.

	Low-Fat Diet (7% Fat)			High-Fat Diet (30% Fat)	
	Soybean Oil (LS)	Perilla Oil (LP)	Olive Oil (LO)	Perilla Oil (HP)	Olive Oil (HO)
β-Cornstarch	39.75	39.75	39.75	16.75	16.75
α-Cornstarch	13.2	13.2	13.2	13.2	13.2
Casein	20.0	20.0	20.0	20.0	20.0
Soybean oil	7.0	–	–	–	–
Perilla oil	–	7.0	–	–	30.0
Olive oil	–	–	7.0	30.0	–
Sucrose	10.0	10.0	10.0	10.0	10.0
Cellulose	5.0	5.0	5.0	5.0	5.0
Vitamin mixture	1.0	1.0	1.0	1.0	1.0
Mineral mixture	3.5	3.5	3.5	3.5	3.5
l-Cysteine	0.30	0.30	0.30	0.30	0.30
Choline bitartrate	0.25	0.25	0.25	0.25	0.25
t-Butylhydroquinone	0.0014	0.0014	0.0014	0.0014	0.0014
Energy (kcal/g)	3.95	3.96	3.95	5.13	5.13

**Table 2:** Primers used for RT-PCR reaction.

Primer		Sequences (5′→3′)
*Elovl2*	Forward	GAGAAGGTGATGTCCGGGTAG
	Reverse	ACATGGACGCGTGGTGATAG
*Elovl5*	Forward	TTCCTCTTGCATCGCGGCT
	Reverse	CCATCCTTTGACTCTTGTATCTCGG
*β-actin*	Forward	GTGGGAATGGGTCAGAAGG
	Reverse	GGTCATCTTTTCACGGTTGG

## Data Availability

The original contributions presented in the study are included in the article, further inquiries can be directed to the corresponding authors.
